# Investigating silver- and gold-functionalized electrospun PHBV fibers as dual-action antimicrobial and immunomodulatory biomaterials 

**DOI:** 10.3389/fbioe.2026.1863464

**Published:** 2026-07-28

**Authors:** Antónia Kurillová, Saverio Caporalini, Bahareh Azimi, Alessandra Fusco, Giovanna Donnarumma, Giovanna Batoni, Stefano Linari, Lucie Suchánková, Renata Večeřová, Libor Kvítek, Aleš Panáček, Serena Danti

**Affiliations:** 1 Department of Physical Chemistry, Faculty of Science, Palacký University in Olomouc, Olomouc, Czechia; 2 Department of Translational Research and New Technologies in Medicine and Surgery, University of Pisa, Pisa, Italy; 3 Consorzio Interuniversitario Nazionale per La Scienza e Tecnologia Dei Materiali (INSTM), Florence, Italy; 4 Department of Civil and Industrial Engineering, University of Pisa, Pisa, Italy; 5 Department of Experimental Medicine, University of Campania “Luigi Vanvitelli”, Naples, Italy; 6 Department of Life Sciences, Health and Health Professions, Link Campus University, Rome, Italy; 7 Linari Engineering S.r.l., Pisa, Italy; 8 Department of Microbiology, Faculty of Medicine and Dentistry, Palacký University in Olomouc, Olomouc, Czechia

**Keywords:** antibacterial, electrospinning, gold nanoparticles, nanomaterials, silver nanoparticles

## Abstract

**Introduction:**

The growing prevalence of antibiotic-resistant bacteria highlights the urgent need for innovative antimicrobial materials. In this work, electrospun poly (3-hydroxybutyrate-co-3-hydroxyvalerate) (PHBV) fibers were functionalized with silver and gold nanoparticles (NPs) to combine high biocompatibility with strong antimicrobial activity.

**Methods:**

Silver and gold NPs were synthesized and characterized, confirming their anisotropic structure and high stability. Electrospinning produced uniform PHBV fibers, and surface modification with NPs via chitosan layer-by-layer ensured stable adhesion.

**Results:**

Antibacterial tests demonstrated that, among all, silver-containing samples exhibited complete inhibition of both *Escherichia coli* and *Staphylococcus aureus*, confirming the strong bactericidal effect of AgNPs. Chitosan alone partially inhibited *S. aureus* and didn't affect *E. coli* due to differences in bacterial cell wall architecture. While chitosan modestly contributed to antibacterial activity, adding AgNPs was crucial for broad, effective antimicrobial action. All functionalized fibers exhibited excellent cytocompatibility with human keratinocytes (HaCaT cells), maintaining over 70% metabolic activity. Moreover, cytokine profiling revealed an anti-inflammatory effect, most pronounced in AgNP-functionalized PHBV samples, and upregulation of human β-defensin 2 (HBD-2) in AuNP-functionalized samples, suggesting additional indirect antibacterial mechanisms.

**Discussion:**

The developed PHBV-based fibers functionalized with metallic NPs demonstrated an excellent balance of safety, cytocompatibility, and antibacterial performance, making them promising candidates for biomedical applications that require both inflammation control and antimicrobial protection.

## 1 Introduction

The rise of antibiotic resistance is one of the most pressing global health challenges, driving the search for alternative antimicrobial strategies that do not rely solely on conventional antibiotics (Salam et al., 2023). Among the most promising solutions, nanomaterials have gained significant attention due to their antibacterial properties and versatility in various applications (Kasapgil et al., 2024). In particular, metallic nanoparticles (NPs), such as silver (AgNPs) and gold (AuNPs), have demonstrated high efficacy against a broad spectrum of bacteria, fungi, and viruses, including drug-resistant strains (Rodrigues et al., 2024; Ratia et al., 2022; Balestri et al., 2023). However, integrating these NPs into functional systems requires a targeted approach to ensure controlled release and adequate compatibility with biological tissues (Matić et al., 2023). In this context, biodegradable polymers represent an ideal platform for NPs delivery within topical applications such as wound healing, combining environmental sustainability with biocompatibility (Wawrzyńczak et al., 2024; Milazzo et al., 2020). For instance, polyhydroxyalkanoates (PHAs) are obtained through microbial fermentation; therefore, this polymer family is considered to be environmentally sustainable (Akaraonye et al., 2010). Moreover, it is characterized by good biocompatibility and biodegradability, making it a viable alternative to traditional polymers used in biomedical applications (Gregory et al., 2022; Miu et al., 2022; Misra et al., 2006). The incorporation of AgNPs has the potential to provide sustained antimicrobial activity to PHAs, thereby expanding the range of applications of the material (Kalaoglu-Altan et al., 2021). The antimicrobial action of silver ions (Ag^+^) released by AgNPs has been shown to interfere with the permeability of the bacterial membrane, thereby compromising its integrity and inducing leakage of essential cellular components through interaction with bacterial proteins and inhibiting key enzymes necessary for microbial survival. Additionally, AgNPs have been observed to induce the production of reactive oxygen species (ROS), contributing to further cellular damage. Consequently, DNA replication is interfered with, leading to a progressive inhibition of bacterial growth. AgNPs have demonstrated antibacterial activity against both Gram-positive and Gram-negative bacteria, although their efficacy may vary depending on differences in cell wall structure, membrane permeability, and bacterial surface composition (More et al., 2023; Yin et al., 2020; Rodrigues et al., 2024). Beyond their antimicrobial properties, AgNPs have also been shown to promote epithelialization and exhibit anti-inflammatory effects, rendering them particularly suitable for tissue engineering and regenerative medicine applications by activating cell proliferation to accelerate regeneration (Carvalho-Silva and Reis, 2024; Loza et al., 2014). Despite their remarkable antibacterial properties, AgNPs are also associated with certain limitations that must be considered in medical applications. These primarily concern the release of Ag^+^ ions, which can lead to oxidative stress, mitochondrial dysfunction, membrane damage, inflammatory reactions, and cytotoxic effects. Nanoparticle aggregation, uncontrolled ion release, or long-term accumulation in biological tissues can negatively impact biocompatibility and clinical safety. Therefore, the controlled immobilization of AgNPs in biocompatible polymer matrices represents an important strategy for reducing potential cytotoxic effects while maintaining antibacterial efficacy (Harun-Ur-Rashid et al., 2025; Sati et al., 2025). On the other hand, gold NPs have garnered significant attention due to their potential applications in the biomedical field. Although AuNPs themselves generally exhibit only minimal intrinsic antibacterial activity, they can interact with biological membranes and, thanks to their unique optical and physicochemical properties, are widely used as versatile nanoplatforms for surface functionalization, targeted drug delivery, biosensing, imaging, and photothermal therapy (Eker et al., 2024; Bansal et al., 2020). The combination of PHA with AgNPs and AuNPs thus represents a promising strategy for developing multifunctional materials with enhanced mechanical properties, biocompatibility, and effective antimicrobial activity. Although the extensive group of PHA biopolyesters has excellent biodegradability and compatibility, some of PHA family members face certain limitations, such as low toughness, fragility, limited mechanical properties, and problematic thermal stability (Frank et al., 2023; Naser et al., 2021). Among them, the copolymer poly (3-hydroxybutyrate-co-3-hydroxyvalerate) (PHBHV, or PHBV) offers improved properties, including higher tensile strength, better processability, and reduced brittleness than poly (hydroxybutyrate) (PHB), making it a preferred polymer for use in biomedical applications, such as scaffolds and wound healing materials, especially in the form of fibrous mesh (Policastro et al., 2021; Simó-Cabrera et al., 2024). The key advantages of PHBV fibers used in wound healing are related to the polymer’s gradual bioabsorption within the wound and its morphological similarity to the skin’s extracellular matrix, which promotes cell adhesion, proliferation, and migration during the healing process (De la Ossa et al., 2022). The addition of substances such as keratin, honey, or metallic NPs also enables the incorporation of anti-inflammatory, antimicrobial, or angiogenic functions into the polymer material (Azimi et al., 2025). Lastly, and equally important, a major advantage is that the tunable properties of the material based on changes in the 3-hydroxyvalerate content in the PHBV copolymer allow the mechanics, degradation, porosity, or absorption capacity to be adjusted (Azimi et al., 2020; Pérez-Galván et al., 2025; Khattab et al., 2018; Zheng et al., 2024). Incorporating bioactive metallic NPs into biodegradable polymeric scaffolds can impart them with antimicrobial properties (Karbowniczek et al., 2023; Castro-Mayorga et al., 2014). Utilizing these materials in the medical field can contribute to preventing nosocomial infections, developing antimicrobial coatings for implants and devices, and advancing wound dressings (Braem et al., 2023). Beyond biomedical applications, these materials could also be employed in the packaging industry to reduce microbial contamination and extend the shelf life of food products (Kusuma et al., 2024).

This study investigates the electrospinning of PHBV, a biodegradable copolymer widely explored in the biomedical field for its excellent biocompatibility, non-toxicity, controlled degradation, and suitability for tissue engineering and wound-healing applications (Francis et al., 2011; De la Ossa et al., 2021; Schmidt et al., 2024). The electrospun PHBV fibers were subsequently surface-functionalized with silver and gold NPs via a chitosan-mediated “layer-on-layer” (L-L) approach. The antibacterial activity of the functionalized fibers was assessed against *Staphylococcus aureus* and *Escherichia coli*, while their cytocompatibility was evaluated using human dermal keratinocytes (HaCaT cells). In addition, the expression of selected pro- and anti-inflammatory cytokines was analyzed to assess their potential relevance for advanced wound dressings or implantable materials intended for infection control and inflammation modulation.

## 2 Materials and methods

### 2.1 Materials

Silver nitrate (AgNO_3_) was purchased from Fagron Italia S.r.l., Quarto Maggiore, BO, Italy. Ammonium hydroxide solution (NH_3_, 28%–30%), sodium borohydride (NaBH_4_, ≥98.0%), Hydrazine hydrate (N_2_H_4_, 50%–60%), Gold (III) chloride trihydrate (HAuCl_4_·3H2O, ≥99.9%), L-ascorbic acid (C_6_H_8_O_6_, 99%), Chitosan (medium molecular weight, 75%–85% deacetylated), Poly (3-hydroxybutyrate-co-3-hydroxyvalerate) (PHBV; HV content 8 mol%), Dulbecco’s Modified Eagle Medium (DMEM), penicillin/streptomycin (Pen-Strep; 10,000 penicillin units and 10 mg streptomycin in 1 mL of 0.9% NaCl solution), phosphate-buffered saline (PBS), resazurin dye and MgCl_2_ were bought from Sigma-Aldrich, Milan, Italy. Potassium dihydrogen citrate (KC_6_H_7_O_7_) was acquired from Lachema n.p., Brno, Czech Republic. Tri-sodium citrate dihydrate (C_6_H_5_Na_3_O_7_·2H_2_O, 99.6%) and acetic acid (CH_3_COOH, 99%) were purchased from Lach-Ner Ltd., Neratovice, Czech Republic. Dichloromethane (DCM) and Methanol (MeOH) were bought from Carlo Erba, Rodano, MI, Italy. Absolute ethanol (EtOH) was purchased from BioOptica Milano S.p.A. (Milan, Italy). Immortalized human keratinocytes (HaCaT cell line, BSL-1) were commercially obtained from ATCC-LGC Standards (Milan, Italy). Fetal calf serum and L-glutamine were acquired from Invitrogen (Carlsbad, CA, USA). *Staphylococcus aureus* (CCM 4223, BSL-2) and *E. coli* (CCM 3954, BSL-2) were obtained from the Czech Collection of Microorganisms (Masaryk University, Brno). All the tested microorganisms were stored in cryotubes (ITEST plus, Czech Republic) at −80 °C. Mueller–Hinton agar and broth (Becton, Dickinson, and Company) were used for bacterial cultivation. Physiological saline (0.9% NaCl) was used for bacterial recovery.

### 2.2 Methods

#### 2.2.1 Synthesis and characterization of AgNP and AuNP dispersion

AgNPs were synthesized through a two-step reduction process, where the first step involved the reduction of the complex cation [Ag(NH_3_)_2_]^+^ using sodium borohydride. This reduction led to the formation of spherical AgNP nuclei, which were formed into larger and more stable anisotropic shapes by the weak reducing agent hydrazine. Citrate played an essential role in synthesis as a stabilizer. The synthesis of AgNPs was carried out at room temperature (RT) under constant stirring with a magnetic stirrer. Gradually, 5 mL of AgNO_3_ (5⋅10^−3^ mol L^-1^), 1.25 mL of NH_3_ (0.1 mol L^-1^), 4.1 mL of KC_6_H_7_O_7_ at 1% (w/w), 10.575 mL of deionized water (dH_2_O), 75 μL of NaBH_4_ (1⋅10^−3^ mol L^-1^), and finally 4 mL of N_2_H_4_ (5⋅10^−2^ mol L^-1^) were added to a beaker to prepare a 25 mL dispersion. AuNPs in the shape of stars were also prepared by a two-step reduction process, where NaBH_4_ and L-ascorbic acid acted as reducing agents. Gold nanostars were formed on silver seeds, where AgNO_3_ was reduced by the strong reducing agent NaBH_4_ to small spherical silver seeds in the first step of the synthesis. The synthesis of gold nanostars was carried out at RT, with a total dispersion volume of 25 mL. The following reagents were sequentially added into a beaker: 0.25 mL of AgNO_3_ (1⋅10^−3^ mol L^-1^), 8.675 mL of deionized water (dH_2_O), 75 μL of NaBH_4_ (1⋅10^−3^ mol L^-1^), 6.25 mL of deionized water, 5 mL of HAuCl_4_·3H_2_O (5⋅10^−3^ mol L^-1^), 0.75 mL of trisodium citrate dihydrate (C_6_H_5_Na_3_O_7_·2H_2_O, 1% w/w), and 4 mL of L-ascorbic acid (1⋅10^−3^ mol L^-1^). The size and morphology of the synthesized AgNPs and AuNPs were characterized by transmission electron microscopy (TEM) using a JEM 2010 TEM instrument (Jeol, Tokyo, Japan). A droplet of the water dispersion containing AgNPs or AuNPs was deposited on a carbon-coated copper grid and dried in a vacuum oven at 25 °C for 1 h. The UV/vis absorbance of AgNPs and AuNPs water dispersions was measured using a Specord S 600 spectrophotometer (Analytic Jena, Jena, Germany). For absorbance measurements, dispersion of anisotropic AgNPs was diluted 10 times, and nanostar-shaped AuNPs were diluted 3 times. The zeta potential of both dispersions was measured using electrophoretic mobility measurements with a Zetasizer NanoZS (Malvern, United Kingdom).

#### 2.2.2 Electrospinning PHBV fibers

The applied protocol is based on the work of De la Ossa *et al.* (De la Ossa et al., 2022) with some modifications and was conducted at Linari Engineering S.r.l. (Pisa, Italy). PHBV fibers were produced using a solution of PHBV at a 15% w/w copolymer concentration, dissolved in a solvent system composed of DCM and MeOH at a 10:1 (w/w) ratio. The solution was prepared by determining the final solution mass and calculating the required amounts of PHBV, DCM, and MeOH. The copolymer was dissolved in the solvent system under gentle agitation in a tightly closed bottle overnight at RT. The electrospinning process was conducted using a custom-made setup within a horizontal setup, utilizing the Starter Kit-Aligned 1 (Linari Engineering s.r.l., Pisa, Italy), which included a 5 mL glass syringe equipped with a G21 stainless steel blunt needle. This syringe was placed on a syringe pump that controlled the flow rate of the polymer solution. The prepared PHBV solution was loaded into a glass syringe, with its needle connected to the ground, and the positive terminal of a high-voltage generator (model S1600079, Linari Engineering s.r.l., Italy) was attached to the collector. The electrospinning was performed at a tip-to-collector distance of 30 cm, a flow rate of 0.96 mL h^-1^, and an applied voltage of 30 kV for 15 min. The fibers were collected on two different collectors*: (i)* a planar collector, and *(ii)* a rotating cylindrical stainless-steel collector (80 mm in diameter and 120 mm in length) with “near still”, i.e., 5 revolutions per minute (rpm), to achieve random fiber orientation. The latter configuration was considered to enable a better uniform fiber distribution. Both were covered with aluminum foil, a feeder, and a ground target to collect the fibers with uniform thickness. The ambient conditions were 25.1 °C temperature and 45.5% relative humidity. The morphology of the electrospun fibers was evaluated qualitatively by scanning electron microscopy (SEM) using a FEI FEG-Quanta 450 instrument (Field Electron and Ion Company, Hillsboro, OR, USA). SEM analysis was conducted by capturing images at different magnifications in randomly selected regions, including the center, middle, and edges of the membrane. Fiber size distribution was obtained by applying “ImageJ” (NIH; Version 1.54p) software to analyze SEM micrographs. At least 100 different fibers were counted (*n* = 100). Fiber diameters were measured by detecting the edges and assuming cylindrical geometry.

#### 2.2.3 PHBV fiber functionalization

The functionalization of PHBV fibers by surface application of AgNPs and AuNPs was achieved using an L-L method, as described in the protocol employed by Kalaoglu-Altan *et al.* (Gregory et al., 2022), Biazar *et al.* (Biazar and Heidari Keshel, 2014), and Bilal *et al.* (Bilal et al., 2019), in which the initial loading on the PHBV fibers consisted of a layer of chitosan, a natural, non-toxic, and biocompatible polymer with a cationic nature to be used as a docking system. Initially, chitosan was dissolved in 1% w/v acetic acid water solution to achieve a final chitosan stock solution with a concentration of 0.5% w/v. The mixture was first stirred vigorously for approximately 20 min, then ultrasonicated to completely break down the remaining solid chitosan particles. Before functionalization, the PHBV fibers were meticulously sectioned into 1 × 1 cm^2^ squares and placed in plastic Petri dishes, each containing a different concentration of chitosan solution (0.5% w/v, 0.25% w/v, or 0.1% w/v). A chitosan solution of varying concentrations was added to the squares to thoroughly cover the samples. After 1 h of exposure to the samples, the copolymer fiber meshes with unbound chitosan were carefully rinsed with deionized water (dH_2_O) and placed in clean Petri dishes. AgNPs or AuNPs dispersions were added to each sample and incubated for 1 h. After exposure to the dispersion, the samples were gently rinsed with dH_2_O to remove unbound NPs. Silver and gold NP-functionalized fiber samples using chitosan (C) at 0.5%, 0.25% and 0.1% w/v were labeled as PHBV/AgNP/C5 and PHBV/AuNP/C5, PHBV/AgNP/C2.5 and PHBV/AuNP/C2.5, PHBV/AgNP/C1 and PHBV/AuNP/C1, respectively. After preparation, they were air-dried in the dark at RT.

#### 2.2.4 PHBV fiber characterization

The morphology of the electrospun fibers was evaluated qualitatively by scanning electron microscopy (SEM) using a FEI FEG-Quanta 450 instrument (Field Electron and Ion Company, Hillsboro, OR, USA). SEM analysis was conducted by capturing images at different magnifications in randomly selected regions, including the center, middle, and edges of the membrane. Fiber size distribution was obtained by applying “ImageJ” (NIH; Version 1.54p) software to analyze SEM micrographs. At least 100 different fibers were counted (*n* = 100). Fiber diameters were measured by detecting the edges and assuming cylindrical geometry. Along with SEM, an Energy-Dispersive X-ray Spectroscopy (EDX) analysis was carried out using a QUANTAX XFlash Detector 6–10 (Bruker, Germany) on the NP-functionalized PHBV fibers to confirm the nature of metallic NPs on their surface.

#### 2.2.5 *In vitro* biological tests with dermal keratinocytes

All the prepared PHBV fibers coated with AgNPs and AuNPs, using the three chitosan concentrations (0.5%, 0.25% and 0.1%), were used for the biological assays after overnight disinfection by absolute EtOH, followed by gentle washing in 3× Pen-Strep/PBS solution for 10 min. After disinfection, all samples were thoroughly rinsed three times with PBS to remove any residual EtOH before usage. The biological assays performed *in vitro* using the human dermal keratinocyte (HaCaT cell line) included the evaluation of cell metabolic activity (as a proof of cell viability), and the inflammatory and immune responses, the latter assessed by measuring the expression levels of the human beta-defensin 2 (hBD-2) antimicrobial peptide. Cells were cultured in DMEM supplemented with 10% v/v fetal calf serum, 1% v/v Pen-Strep (i.e., final concentration: 100 U mL^-1^ penicillin and 100 μg mL^-1^ streptomycin) and 1% v/v L-glutamine. HaCaT cells were plated in 96-well plates and incubated at 37 °C in a humidified atmosphere of 95% air and 5% CO_2_ until they reached 80% confluence. Sterile fiber mesh samples, cut with a 6-mm dermal puncher into discs, were added to the cultures and incubated for 24 h before performing metabolic activity and molecular biology assays, using triplicate samples for each test (*n* = 3).

To check cell viability (according to UNI EN ISO 10993–5), resazurin (R), a metabolic dye, was added at a concentration of 0.5 mg mL^-1^ in culture medium and incubated for 4 h. This dye (blue) is reduced by metabolically active cells into resorufin (pink), indicating metabolic activity and cell viability. Briefly, samples, cell-free negative controls, and sample-free cells (positive controls) were incubated for 3 h at 37 °C with Resazurin dye diluted in culture medium according to the manufacturer’s instructions. Then, 100 μL of supernatant taken from the sample or the control was loaded into 96-well plates. The supernatants were analyzed using a spectrophotometer (Victor 3, PerkinElmer, Waltham, MA, USA) with double-wavelength readings at 570 nm and 600 nm. Therefore, the percentage of Resazurin reduction (%R_REDUCED_) was calculated by correlating the absorbance values with the dye’s molar extinction coefficients at the selected wavelengths. The equation applied is shown below (Equation 1):

$$R_{\text{red}}\%=100\times \frac{117,216\;‧A\left(\lambda_{570\;\text{nm}}\right)-80,586\;‧A\left(\lambda_{600\;\text{nm}}\right)\;}{\;155,677‧A{}^{\circ}\left(\lambda_{600\;\text{nm}}\right)-14,652\;‧A{}^{\circ}\left(\lambda_{570\;\text{nm}}\right)\;}$$ (1)

where A is the absorbance value of the samples and A° is the absorbance value of the negative control, the latter indicating the dye-conditioned medium incubated in the presence of fiber mesh without any cells.

The *in vitro* inflammatory and immune responses were investigated by measuring the mRNA expression levels in HaCaT cells seeded in direct contact with the functionalized fiber meshes. After 6 h and 24 h of incubation, mRNA was extracted from the cells. The expression levels of pro-inflammatory cytokines, including Interleukins (ILs) IL-1α, IL-1β, IL-6, IL-8, and Tumor Necrosis Factor α (TNF-α), the anti-inflammatory cytokine Transforming Growth Factor β (TGF-β), and the antimicrobial peptide HBD-2 were evaluated by quantitative real-time polymerase chain reaction (qRT-PCR). Briefly, the total RNA was isolated with TRizol, and 1 µL of RNA was reverse transcribed into complementary DNA (cDNA) using random hexamer primers at 42 °C for 45 min, according to the manufacturer’s instructions. RT-PCR was carried out with the LC Fast Start DNA Master SYBR Green kit using 2 µL of cDNA corresponding to 10 ng of total RNA in a 20 µL final volume, 3 mM MgCl_2_, and 0.5 µM sense and antisense primers (Table 1). The results were normalized by the expression of the same cytokine in untreated cells, as a control (Ctrl).

**TABLE 1:** QRT-PCR primer sequence and operating conditions for HaCaT cells.

Gene	Primer sequence (Forward and reverse)	Conditions	Base pairs
IL−1α	5′−CATGTCAAATTTCACTGCTTCATCC−3′ 5′−GTCTCTGAATCAGAAATCCTTCTATC−3′	5 s at 95 °C, 8 s at 55 °C, 1 s at 72 °C for 45 cycles	421
IL−1β	5′−GCATCCAGCTACGAATCTCC−3′ 5′−CCACATTCAGCACAGGACTC−3′	5 s at 95 °C, 14 s at 58 °C, 28 s at 72 °C for 40 cycles	708
TNF−α	5′−CAGAGGGAAGAGTTCCCCAG−3′ 5′−CCTTGGTCTGGTAGGAGACG−3′	5 s at 95 °C, 6 s at 57 °C, 13 s at 72 °C for 40 cycles	324
IL−6	5′−ATGAACTCCTTCTCCACAAGCGC−3′ 5′−GAAGAGCCCTCAGGCTGGACTG−3′	5 s at 95 °C, 13 s at 56 °C, 25 s at 72 °C for 40 cycles	628
IL−8	5−ATGACTTCCAAGCTGGCCGTG−3′ 5−TGAATTCTCAGCCCTCTTCAAAAACTTCTC−3′	5 s at 94 °C, 6 s at 55 °C, 12 s at 72 °C for 40 cycles	297
TGF−β	5’-CCGACTACTACGCCAAGGAGGTCAC-3’ 5’-AGGCCGGTTCATGCCATGAATGGTG-3’	5 s at 94 °C, 9 s at 60 °C, 18 s at 72 °C for 40 cycles	439
HBD−2	5′−GGATCCATGGGTATAGGCGATCCTGTTA−3′ 5′−AAGCTTCTCTGATGAGGGAGCCCTTTCT−3′	5 s at 94 °C, 6 s at 63 °C, 10 s at 72 °C for 50 cycles	198

#### 2.2.6 *In vitro* antibacterial tests

AgNP-functionalized samples were investigated for antibacterial activity, following the method outlined in ISO 22196:2007(E). Test inoculum of *Staphylococcus aureus* (CCM 4223) and *Escherichia coli* (CCM 3994) were prepared in 1/500 nutrient broth to a concentration of 6·10^5^ cells/mL. A 0.1 mL aliquot of this bacterial suspension was pipetted onto 25 × 25 mm^2^ treated and untreated (control) test specimens, and the specimens were covered with 20 × 20 mm^2^ sterile plastic film. The inoculated specimens were then incubated for 24 h at 37 °C and a relative humidity greater than 90%. Following incubation, surviving bacteria were recovered by washing the surface with 5 mL of physiological saline (0.9% NaCl). A 100 µL aliquot of the wash solution was transferred to agar plates and spread evenly. Plates were incubated for 24 h at 37 °C, prior colony forming unit (CFU) counting. For each specimen, the number of viable bacteria recovered per square centimeter (N) was calculated using the following equation (Equation 2), as from ISO 22196:

N=100×C×D×V×A (2)

where: N = number of viable bacteria per cm^2^; C = average plate count from duplicate Petri dishes at a specific dilution; D = dilution factor for the plates that were counted; V = volume (in mL) of neutralizer solution used to recover bacteria from the specimen (i.e., 5 mL); A = surface area of the cover film in mm^2^ (400 mm^2^); 100 = conversion factor from mm^2^ to cm^2^. The number of viable bacteria recovered per cm^2^ was calculated for each replicate specimen (*n* = 3), and the geometric mean of these values was determined and expressed to two significant figures. In cases where no colonies were recovered, the number of colonies was recorded as “< 1” and N was recorded as “< V”. For the calculation of the geometric mean, these specimens were considered equal to V to avoid computational errors. The resulting N values were used to determine U_0_ (time-zero control), U_t_ (untreated specimen after 24 h), and A_t_ (treated specimen after 24 h), which in turn were used to calculate the antibacterial activity, R, as R = U_t_ − A_t_.

#### 2.2.7 Statistical analysis

Statistical analysis was conducted using SPSS software. Data are given as mean ± standard deviation (SD). Differences between groups were analyzed using one-way ANOVA followed by Tukey’s *post hoc* test. Significance was determined at a probability *p* < 0.05.

## 3 Results

### 3.1 Synthesis of anisotropic Ag and Au NPs

**FIGURE 1:**
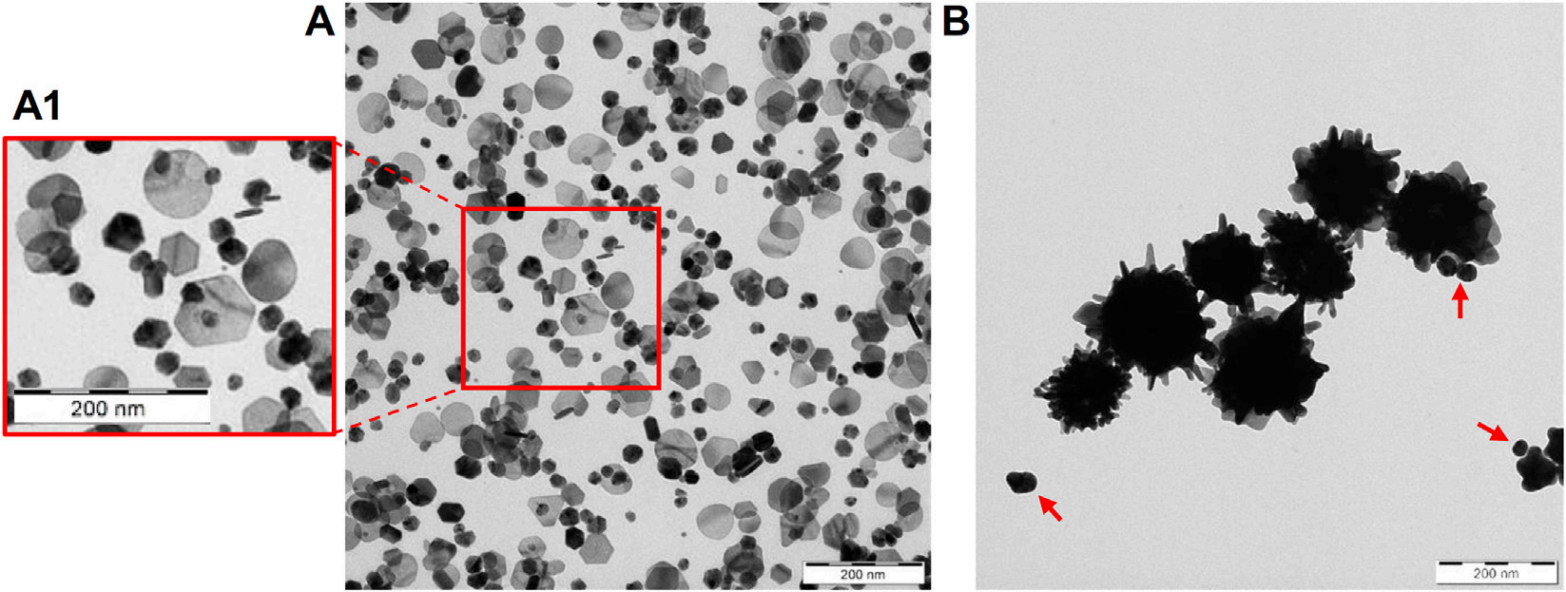
TEM micrographs showing anisotropic shapes obtained in: **(A)** AgNPs with a wide distribution of plate-like structures, nanorods and small spherical structures; (A1) lens at higher magnification; and **(B)** larger nanostar-like AuNPs together with smaller spherical AuNPs, pointed out with arrows.

AgNPs and AuNPs were successfully synthesized via a two-step reduction method designed to induce anisotropic growth. The morphology and size of the synthesized NPs were characterized by TEM, while their optical properties and stability were evaluated using UV-Vis spectroscopy and zeta potential measurements. TEM analysis confirmed that the synthesis protocol yielded particles with distinct anisotropic shapes (Figure 1). For AgNPs, the reduction of [Ag(NH_3_)_2_]^+^ using hydrazine as a secondary reducing agent facilitated the transformation of spherical nuclei into larger, mostly plate-like, and faceted structures. These AgNPs exhibited a size distribution ranging from 40 to 60 nm. In the case of AuNPs, the use of ascorbic acid and sodium borohydride resulted in the formation of “nanostars”, namely, particles with a central core and multiple branching spikes. These Au nanostars were significantly larger, with an average size of approximately 200 nm.

**FIGURE 2:**
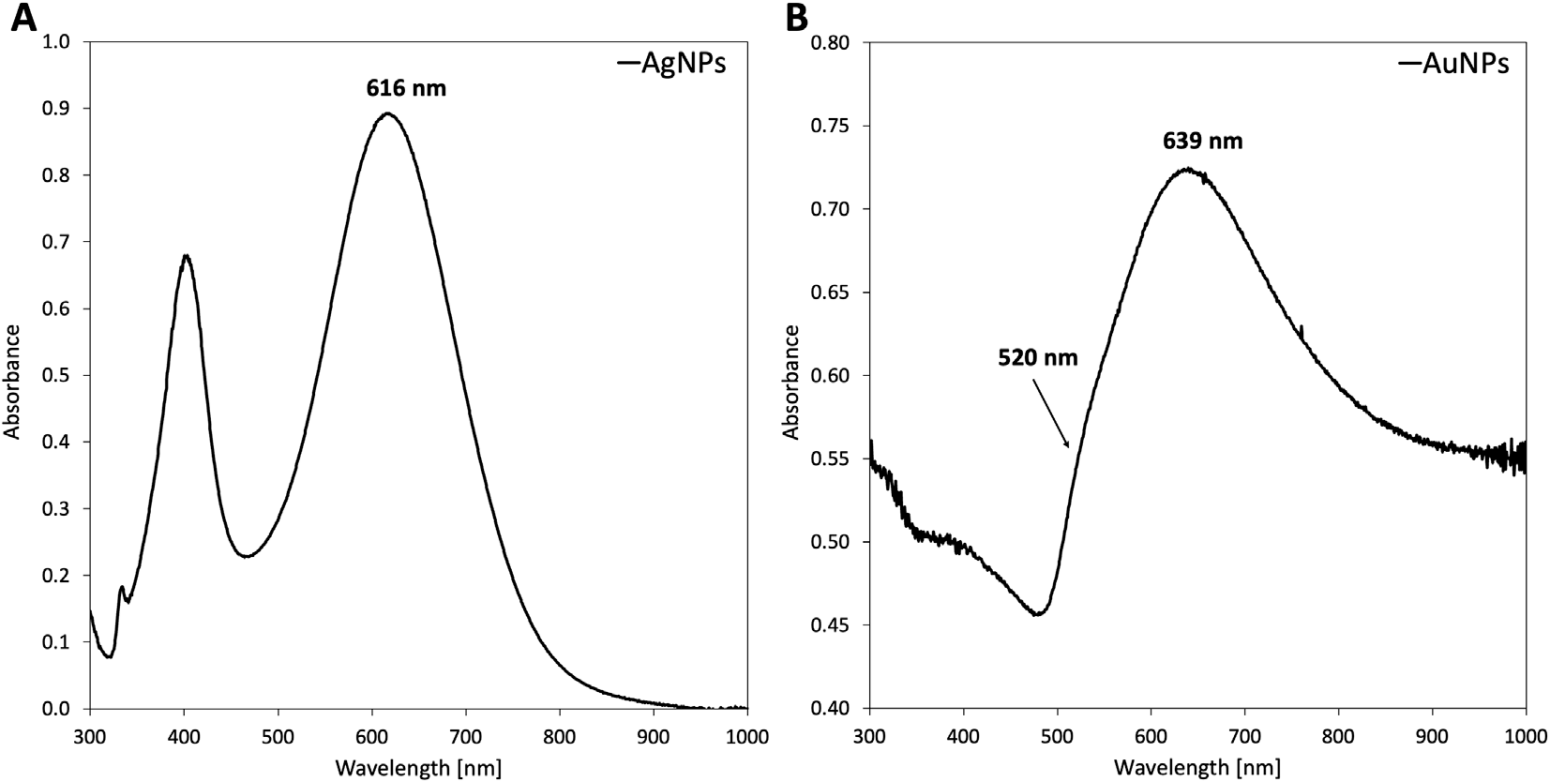
UV/vis spectra of **(A)** AgNPs, and **(B)** AuNPs.

The anisotropic nature of the NPs was further corroborated by UV-Vis spectroscopy (Figure 2). The AgNP dispersion displayed a distinct absorption peak at 616 nm. This represented a significant red shift compared to the typical plasmon resonance of spherical AgNPs (usually ∼400 nm), effectively confirming the presence of plate-like or faceted morphologies, which alter the localized surface plasmon resonance (LSPR) modes. The spectrum also exhibited a distinct absorption band with a maximum at approximately 402 nm (Figure 2). This peak is typical for surface plasmon resonance (SPR) of silver nanospheres, indicating the coexistence of spherical silver NPs, but their occurrence is markedly smaller compared to anisotropic structures. This interpretation is directly supported by TEM images (Figure 1). Similarly, AuNPs exhibited a broad absorption peak centered at 639 nm. This value is considerably higher than the characteristic ∼520 nm peak observed for spherical gold colloids, supporting the formation of star-shaped structures with extended tips that shift the LSPR to longer wavelengths. A weak shoulder around 520 nm is discernible in Figure 2B, corresponding to the localized surface plasmon resonance of spherical gold NPs, indicating that their presence in the dispersion is minor.

The stability of the nanoparticle dispersions was assessed via zeta potential measurements. AgNPs and AuNPs exhibited zeta potential values of −22.2 mV and −34.5 mV, respectively. These strongly negative values indicate that the citrate ions used during synthesis effectively acted as stabilizing agents, creating enough electrostatic repulsion between particles to prevent aggregation and ensure the sufficient stability of the dispersion during storage and handling prior to immobilization, while also facilitating strong electrostatic interactions with positively charged chitosan molecules during the modification of PHBV fibers.

### 3.2 PHBV fiber preparation and functionalization

**FIGURE 3:**
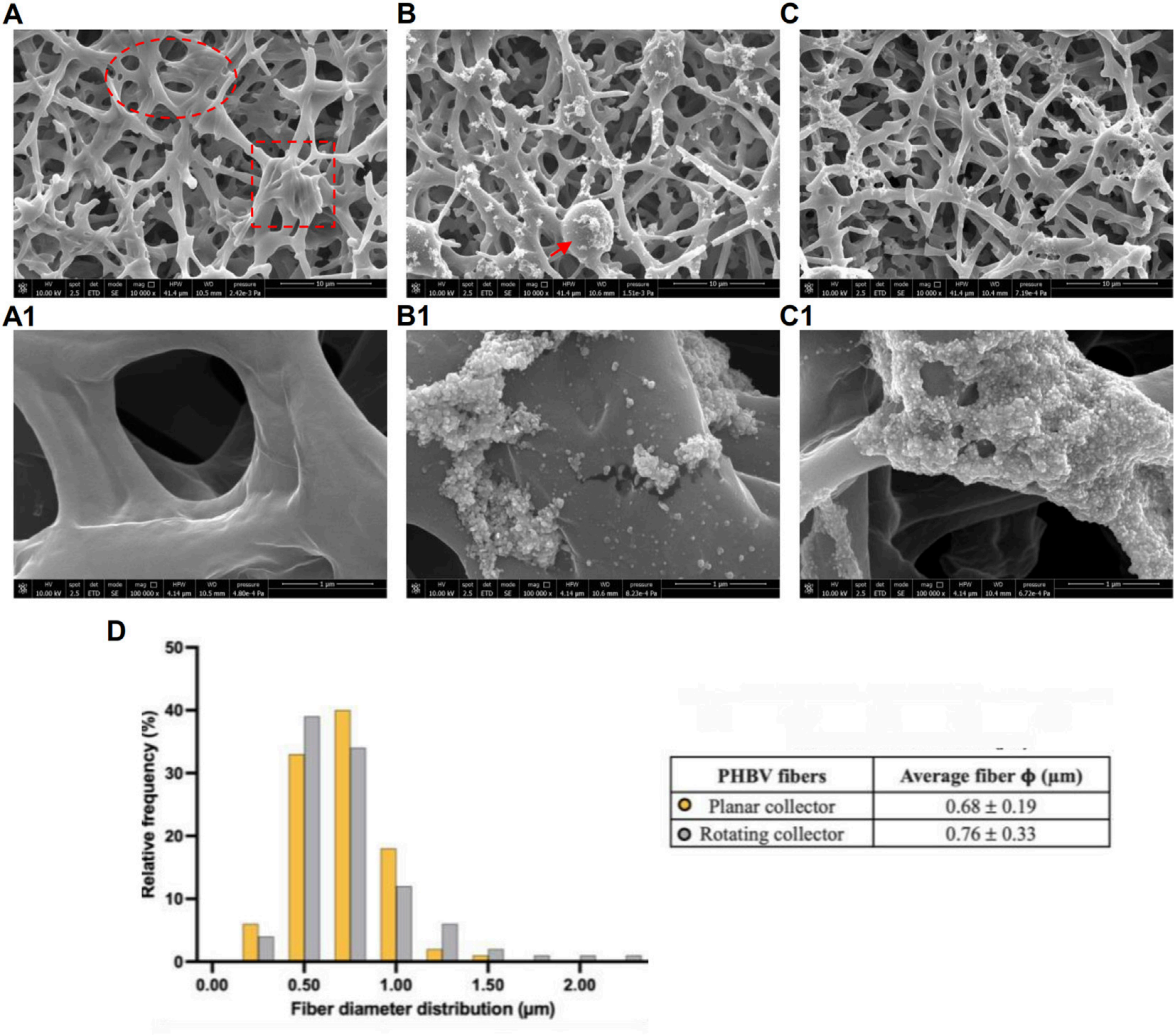
Morphological analysis obtained via SEM imaging of: (A, A1) plain, (B, B1) AgNP-chitosan functionalized, and (C, C1) AuNP-chitosan functionalized PHBV fiber meshes, showing: **(A–C)** fiber mesh morphology, and (A1-C1) fiber surfaces. **(D)** PVHB fiber size distribution collected with a planar or near-zero rotating collector. **(A–C)** SEM micrographs were acquired at 10 kV and 10,000× magnification; **(A)** Dashed areas show (circle) melted or (square) agglomerated fibers; **(B)** arrow points to a bead. (A1-C1) SEM micrographs were acquired at 10 kV and 100,000× magnification; (A1) smooth PHBV fiber surface; (B1, C1) PHBV fiber surface decorated with chitosan embedding: (B1) AgNPs, and (C1) AuNPs.

**FIGURE 4:**
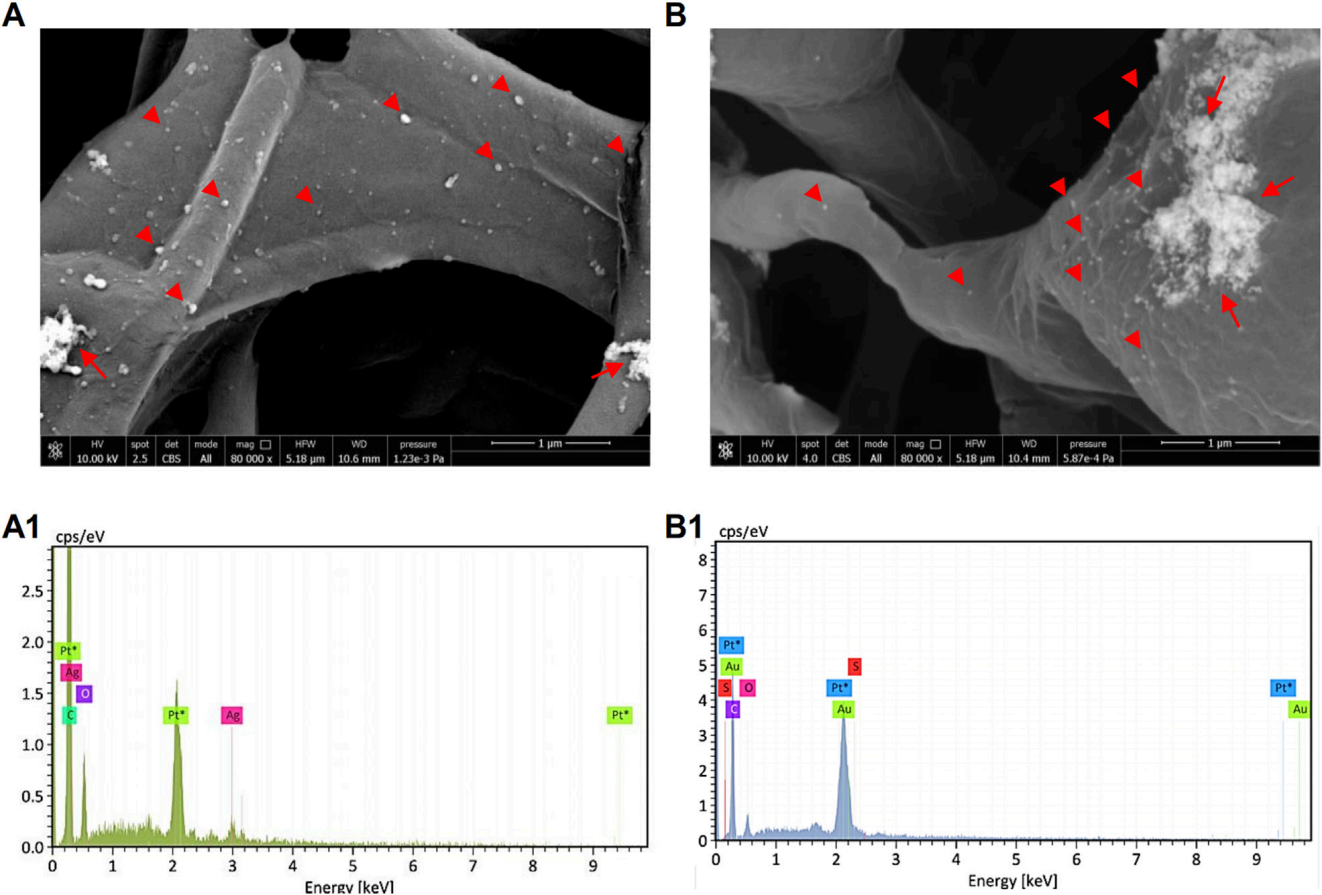
**(A,B)** SEM micrographs showing single and aggregated particles adhered to the PHBV fiber surfaces, which display small wrinkle due to the presence of chitosan as a binding agent; **(A)** AgNPs, and **(B)** AuNPs. SEM micrographs were acquired at 10 kV and 80,000× magnification; Arrows point to NP aggregates, while arrowheads indicate some of the single NPs. (A1, B1) EDX analysis showing the distinctive elements present on the sample surface, namely, (A1) Ag, and (B1) Au. The presence of Pt is due to the sputter-coating agent.

The morphology of the electrospun PHBV fibers was analyzed using SEM to evaluate the effectiveness of the electrospinning process parameters. SEM micrographs revealed that the resulting fibers had a smooth, branched morphology (Figure 3A, A1). Fibers formed randomly oriented non-woven webs both on a planar and a rotating collector set to a “near 0 rpm” rotation speed. While the fiber network was generally continuous, some morphological defects, specifically bead formation, melted or agglomerated fibers, were observed scattered throughout the sample (Figure 3A, B). Image analysis showed that the PHBV fibers possessed a wide diameter distribution ranging from 0.25 to 2.50 μm; however, the highest frequency was confined to 0.50–1.00 μm, with minimal difference in fiber size distribution using both collectors (Figure 3D). By using chitosan as a primer, both AgNPs and AuNPs could be deposited on the PHBV fibers without relevant modification of the fiber mesh structure (Figure 3B, B1, C, C1). While the plain PHBV fibers exhibited a smooth surface with occasional beads, the functionalized samples displayed a distinct surface roughness attributed to the dense coating of metallic NPs (Figure 4 A1-C1).

The SEM micrographs reveal a non-uniform distribution of metal NPs on the surface of the PHBV fibers. Instead of a continuous, homogeneous coating, particles tend to form distinct aggregates irregularly scattered along the fiber network. The formation of these aggregates on the PHBV fibers can be attributed to the high surface energy of the NPs, which drives them to minimize their total surface area by clustering during the deposition/electrospinning process (Figure 4 A, B). Although the exact thickness of the chitosan layer was not directly measured, its ultrathin (nanometer-scale) nature is indirectly demonstrated by the SEM analysis. As documented in Figure 4, the introduction of chitosan did not alter the distinct fibrous morphology, interconnectivity of the PHBV matrix. A thicker polymeric coating would inevitably lead to significant fiber thickening, pore clogging, or film formation at the fiber intersections, none of which were observed here. The assumption of an ultrathin chitosan coating is further supported by the deposition parameters. The use of a highly diluted chitosan solution ensures a low-viscosity medium that forms a conformal, sub-micron layer rather than a bulk coating. To validate the chemical composition of the deposited layers, EDX was performed on the functionalized samples. The EDX spectra clearly showed strong signals corresponding to elemental silver (Ag) and gold (Au) for the respective samples (Figure 4 A1, B1). This confirms that the particles observed in the SEM micrographs are indeed metallic NPs and demonstrates the effectiveness of the chitosan-mediated L-L strategy for activating the surface of electrospun PHBV fibers.

### 3.3 Biological evaluations

**FIGURE 5:**
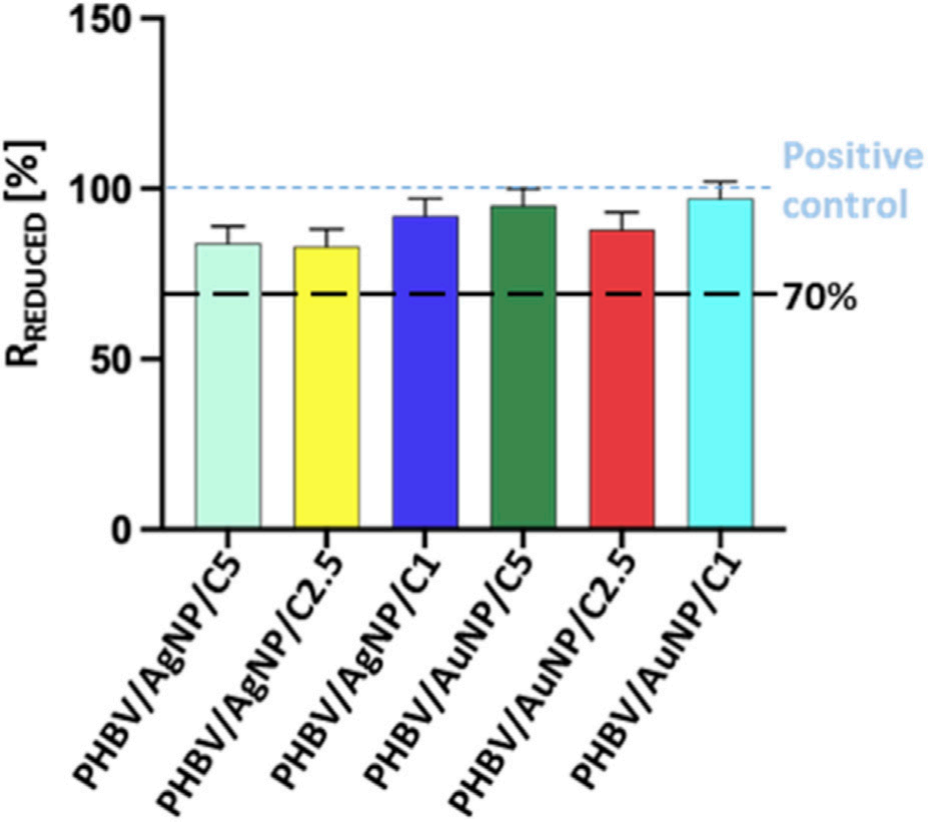
Results of metabolic activity of the HaCaT cell line after 24 h cultured on PHBV/AgNPs and PHBV/AuNPs with different chitosan (C) concentrations, namely, 0.5% w/v (C5), 0.25% w/v (C2.5) and 0.1% w/v (C1) (*n* = 3), indicating cell viability as Resazurin dye reduction percent (R_REDUCED_); All the fiber meshes were highly cytocompatible (R_REDUCED_ > 70%); the value of the positive control (sample-free HaCaT cells) is set at 100%.

The cytocompatibility of functionalized PHBV fibers was evaluated using HaCaT cells. The results of cytocompatibility, presented in Figure 5, demonstrated the metabolic activity of the HaCaT cell line exposed to the samples.

**FIGURE 6:**
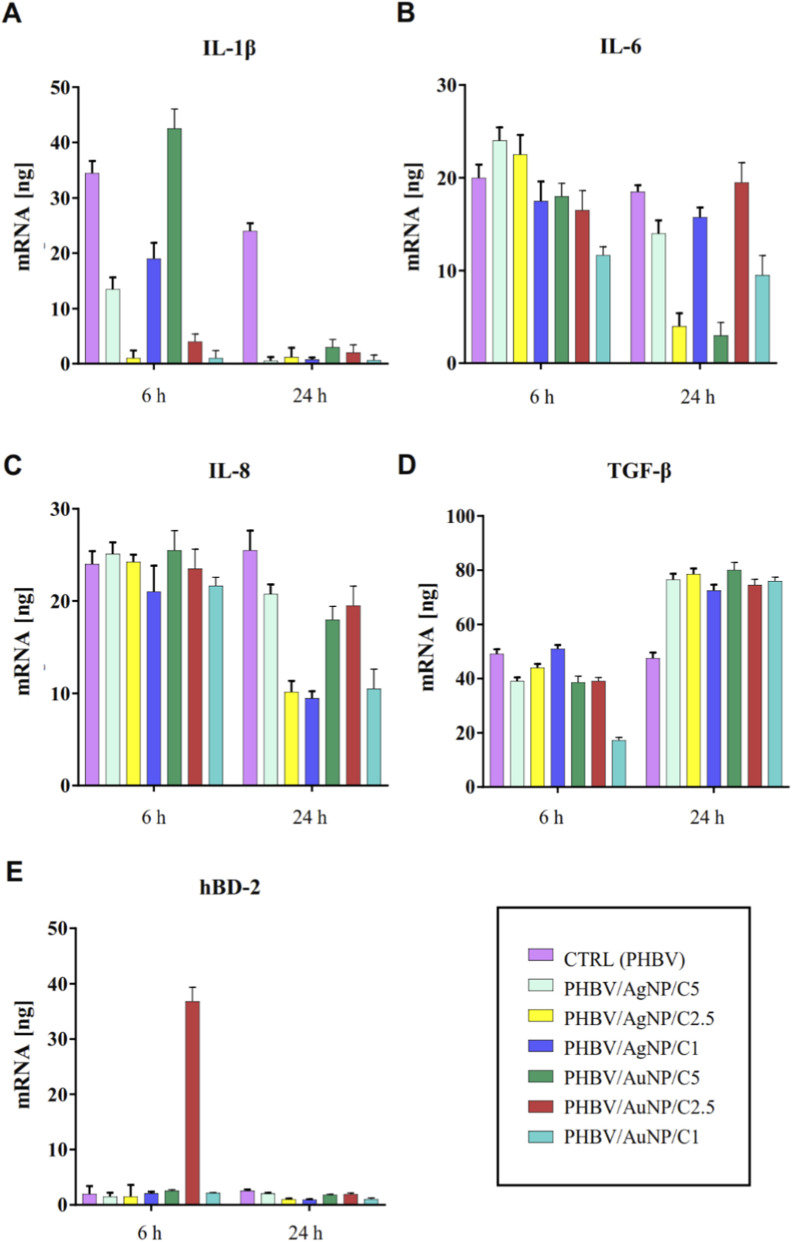
The bar graphs display the mRNA expression levels in HaCaT cells of **(A-C)** pro-inflammatory cytokines IL-1β, IL-6 and IL-8, **(D)** anti-inflammatory cytokine TGF-β, and **(E)** antimicrobial peptide hBD-2, for the different samples produced (*n* = 3) at two time-points: 6 h and 24 h. CTRL = Control (HaCaT cells exposed to plain PHBV fibers); untreated cells were used as basal reference for normalization.

The Resazurin assay demonstrated that all samples maintained metabolic activity values exceeding 70% compared with untreated positive controls (100%), showing excellent cytocompatibility. No statistically significant influence of chitosan concentration was observed. To evaluate the bioactive potential of the functionalized fibers beyond antibacterial effects, cytokine expression analysis was performed on HaCaT cells exposed to the materials for 6 h and 24 h (Figure 6). Keratinocytes are highly responsive epithelial cells involved in the early phases of cutaneous injury and repair, and their contact with electrospun fibrous scaffolds may induce transient inflammatory signaling associated with early cell–material interaction. The transient increase in IL-1β expression observed after 6 h in cells cultured on untreated PHBV fibers may reflect an early cellular response to direct contact with the fibrous scaffold rather than long-term inflammatory activation. This study was not designed to elucidate the underlying mechanism; therefore, it is not possible to determine whether this response is related to the intrinsic responsiveness of HaCaT cells under the selected culture conditions, the scaffold topography, surface chemistry, or other material-related factors. Although it has been reported that PHBV exhibits electroactive or piezoelectric properties, the experiments were conducted under static culture conditions without applied mechanical stimulation, and thus, no conclusions can be drawn regarding the contribution of piezoelectric effects. However, the contribution of remnant polarization or electroactive behavior under static conditions cannot be completely excluded, as similar phenomena have been reported in electroactive polymer fiber systems even without external mechanical loading (Azimi et al., 2024). Likewise, since all experimental groups were cultured under identical conditions, the observed response cannot be attributed to differences in calcium concentration in the culture medium. Among the possible material-related factors, the architecture of the micro-/nanofibrous scaffold may influence early cell-material interactions; however, this aspect was not directly investigated in this study. Importantly, IL-1β expression decreased significantly after 24 h, while samples functionalized with nanoparticles generally exhibited lower expression levels than pure PHBV, suggesting that surface functionalization modulates the initial cellular response to the scaffold (Milazzo et al., 2020).

At the endpoint, all functionalized fibers induced a clear anti-inflammatory profile characterized by downregulation of pro-inflammatory cytokines, in particular IL-1β, which was markedly reduced by all the functionalized samples and not by the plain PHBV, but also IL-6 and IL-8 were reduced by some coformulations, i.e., PHBV/AgNP/C2.5, PHBV/AuNP/C5 and PHBV/AuNP/C1. After 24 h, all the functionalized samples, but not the plain PHBV, induced upregulation of the anti-inflammatory cytokine TGF-β. Among the tested samples, PHBV/AgNP/C2.5 exhibited the strongest effect across the diverse cytokines tested, inducing a drop in IL-1β already 6 h after contact, alongside reductions in the other pro-inflammatory cytokines and the concomitant upregulation of the anti-inflammatory TGF-β. Furthermore, PHBV/AuNP/C2.5 notably upregulated the antimicrobial peptide hBD-2 after 6 h, suggesting that AuNPs may indirectly enhance innate immune response. Other formulations, such as PHBV/AuNP/C1, showed a similar anti-inflammatory pathway for the investigated cytokines, but not for hBD-2.

**FIGURE 7:**
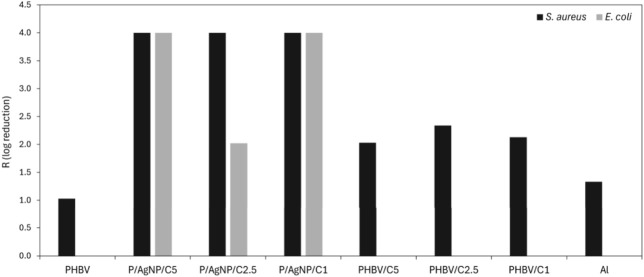
Antibacterial activity of functionalized polymer samples expressed as R (log reduction) according to ISO 22196, after 24 h of incubation with *S. aureus* and *E. coli*. PHBV coated with AgNPs using 0.5% and 1% chitosan (C) solutions showed the highest antibacterial activity, achieving an R value of 4 (>99% reduction in viable cells) for both bacterial species. Without AgNPs, PHBV/C exhibited only a slight effect against *S. aureus* and no detectable activity against *E. coli*. Control (plain PHBV) and aluminum substrate (Al) did not show notable antibacterial activity; *n* = 3.

Since free NPs were not tested as independent controls, the observed cellular responses should be interpreted as the effect of the combined functional material, where AgNPs or AuNPs are immobilized on the PHBV/chitosan fibrous surface, rather than as the response to isolated nanoparticle exposure. While both silver and gold NPs were integrated into the polymer system and their cytocompatibility and inflammatory responses were assessed, antibacterial testing was conducted only on samples with silver NPs. This choice stemmed from silver’s established and clinically validated broad-spectrum antimicrobial properties, whereas gold NPs are predominantly valued for their biocompatibility rather than strong antibacterial effects. As a result, the antibacterial evaluation focused on the material version, most likely to exhibit effective antimicrobial activity. The antibacterial activity of the PHBV/AgNP formulations was evaluated according to ISO 22196:2007(E), and antibacterial performance was expressed as the antibacterial activity value (R), calculated from the logarithmic difference between viable counts recovered from untreated control specimens (U_t_) and treated specimens (A_t_) after 24 h incubation (see Method section). Pronounced differences in antibacterial efficacy were observed among the tested surfaces (Figure 7). The PHBV/AgNP/C1 and PHBV/AgNP/C5 formulations exhibited the highest antibacterial activity against both *Escherichia coli* and *Staphylococcus aureus*, reaching an R value of 4.0 after 24 h. Under the experimental conditions applied, no viable bacteria were recovered from these surfaces. Similarly, PHBV/AgNP/C2.5 achieved an R value of 4.0 against *S. aureus*, indicating complete eradication of recoverable viable cells. In contrast, PHBV/AgNP/C2.5 showed a lower antibacterial effect against *E. coli*, with an R value of approximately 2.0, corresponding to a 99% reduction in viable bacteria compared with the untreated control. The pristine PHBV copolymer demonstrated limited antibacterial activity, yielding an R value of approximately 1.0 against *S. aureus* and no measurable antibacterial effect against *E. coli* (R = 0). Chitosan-modified PHBV surfaces (0.1%–0.5% w/v, without AgNPs) did not exhibit antibacterial activity against *E. coli* but showed moderate activity against *S. aureus*, with R values up to approximately 2.5 depending on chitosan concentration. The aluminum reference substrate exhibited similar modest antibacterial performance, with an R value of approximately 1.3 against *S. aureus* and no detectable effect against *E. coli*. The low but measurable reduction values observed for both pure PHBV (R = 1) and the aluminum substrate against *S. aureus* are more likely related to the physical properties of the surface than to intrinsic chemical bactericidal properties. Hydrophobic polymer matrices, such as PHBV, may partially limit the initial attachment of bacteria, while the non-porous surface of the aluminum collector may contribute to a partial loss of bacterial viability under the limited conditions of the 24-h incubation protocol according to ISO 22196. Aluminum was included in the antibacterial tests because it served as the collector’s base substrate during the electrospinning process, thereby ensuring that the material’s base components did not artificially contribute to the high antibacterial efficacy demonstrated by silver-containing systems.

## 4 Discussion

This study demonstrates the successful development of multifunctional electrospun PHBV fiber meshes surface-functionalized with anisotropic silver and gold NPs through a chitosan-mediated layer-on-layer strategy. The resulting materials combined structural integrity, antibacterial efficacy, cytocompatibility, and immunomodulatory activity, thereby highlighting their potential for advanced wound-healing and implant-related applications. The synthesis approach applied in our study yielded anisotropic AgNPs and AuNPs with optical signatures consistent with their non-spherical morphologies. The red-shifted plasmon resonance observed for AgNPs at 616 nm, together with a secondary band around 402 nm, confirmed the coexistence of platelet-like/faceted structures and a minor population of spherical NPs, as directly evidenced by TEM. Similarly, the broad absorption band of AuNPs centered at 639 nm is characteristic of nanostar geometries, where sharp tips and branched morphologies support multiple localized surface plasmon resonance modes (Dobrynin et al., 2025). These anisotropic features are particularly relevant in biomedical contexts, as they increase surface area and enhance interfacial interactions with biological environments compared to spherical counterparts.

Electrospun PHBV fibers were successfully produced using a DCM/MeOH solvent system, which led to nonwoven meshes with fiber diameters predominantly in the submicron-to-micron range. Although some bead formation and localized fiber fusion were observed, these morphological features are commonly reported for PHBV electrospinning and can be attributed to the interplay between solvent volatility, polymer concentration and jet stability (De la Ossa et al., 2022; Caporalini et al., 2025). Importantly, the resulting fibrous architecture provides a high surface-to-volume ratio and morphological similarity to the native extracellular matrix, which is advantageous for both surface functionalization and cell–material interactions in wound healing applications (Kalaoglu-Altan et al., 2021). Surface functionalization was achieved without compromising fiber morphology by exploiting electrostatic interactions between negatively charged citrate-stabilized NPs and a positively charged chitosan interlayer. Indeed, the stability and functionality of complex multilayer systems, including hybrid hydrogel coatings containing PHBV fibers, depend on the selection of efficient primers that modulate physicochemical and mechanical properties (Cristallini et al., 2019). The layer-on-layer strategy was successfully applied to overcome the chemical inertness and hydrophobicity of PHBV, enabling stable NP immobilization at the surface rather than in the bulk. SEM and EDX analyses confirmed that both AgNPs and AuNPs were predominantly localized at the fiber surface, where they remained accessible to the surrounding biological environment. This surface-specific exposure is essential for antibacterial activity and for modulating cell responses while minimizing uncontrolled NP release (Le Ouay and Stellacci, 2015; Bruna et al., 2021).

From a biological standpoint, all functionalized fibrous meshes exhibited excellent cytocompatibility toward human keratinocytes, with metabolic activity consistently exceeding the 70% threshold defined by ISO 10993–5 standards. Overnight disinfection with EtOH should be considered a relevant processing step rather than a completely neutral handling procedure. Although ethanol may affect colloidal stability and surface interactions in dispersions, particularly those of silver nanoparticles, in this system, the nanoparticles were immobilized within a chitosan-mediated layer-by-layer architecture on PHBV fibers, which should significantly limit extensive rearrangement of the nanoparticles. Under these conditions, AuNPs are expected to remain chemically stable, whereas for AgNPs, subtle changes in the outer weakly bound surface components cannot be completely excluded. Importantly, biological tests were performed following identical EtOH/PBS treatment, and the preserved activity suggests that the functional surface properties of the fibers were maintained after disinfection (Kaul et al., 2025; Liz-Marzán and Lado-Touriño, 1996; Chowdhury et al., 2025; Shutava and Livanovich, 2020). The absence of significant cytotoxic effects across all chitosan concentrations suggests that the chitosan-mediated immobilization effectively moderates NP bioavailability, preventing excessive metal ion release. The slight reductions in metabolic activity observed for some AgNP-containing samples remained within acceptable limits and are consistent with literature reports describing a balance between antimicrobial efficacy and host cell tolerance when silver is present in a controlled, surface-bound form (Akter et al., 2018). Beyond cytocompatibility, the developed materials exhibited distinct and formulation-dependent immunomodulatory effects. Notably, all NP-functionalized PHBV fibers induced a pronounced anti-inflammatory profile, characterized by the downregulation of key pro-inflammatory cytokines such as IL-1β, IL-6, and IL-8, alongside the upregulation of the anti-inflammatory cytokine TGF-β at 24 h, highlighting the prominent role of PHBV electrospun fibers in this context (De la Ossa et al., 2021; Reay et al., 2024). Among the tested conditions, PHBV/AgNP/C2.5 emerged as the most effective formulation, eliciting an early suppression of IL-1β and a coordinated modulation of inflammatory mediators. These findings aligned with growing evidence that controlled silver ion exposure can attenuate excessive inflammation while supporting tissue regeneration, a critical requirement for chronic wound management (Ninan et al., 2020). Interestingly, AuNP-functionalized fibers displayed a complementary biological role. PHBV/AuNP/C2.5 notably upregulated the antimicrobial peptide human β-defensin 2 (hBD-2) at early time points. HBD-2 is a key component of the epithelial innate immune response, exerting antimicrobial effects and acting as a chemoattractant for immune cells (Donnarumma et al., 2016). This suggests that AuNPs may enhance host defense mechanisms indirectly by stimulating endogenous antimicrobial pathways rather than acting as direct antimicrobial agents. Such immunoprotective behavior is particularly valuable in wound healing, where balanced immune activation is essential to prevent infection without triggering chronic inflammation (Dykman and Khlebtsov, 2017).

The antibacterial testing performed according to ISO 22196 revealed pronounced differences among the evaluated surfaces. For both *E. coli* and *S. aureus*, samples containing silver completely inhibited bacterial growth, corresponding to a reduction of approximately four orders of magnitude compared with the time-zero control. In contrast, the pristine polymer and the polymer surfaces modified solely with chitosan did not demonstrate a strong reduction in *E. coli* counts, indicating that chitosan alone did not exert detectable inhibitory effects under these conditions. For *S. aureus*, chitosan-modified polymer surfaces contributed to a moderate reduction in viable counts of about one order of magnitude, suggesting that chitosan itself had a partial inhibitory effect against *S. aureus*. The observed higher susceptibility of *S. aureus* compared with *E. coli* to chitosan can be attributed to structural differences in their cell envelopes. Chitosan exerts antimicrobial activity through electrostatic interactions between its positively charged amino groups and negatively charged bacterial surface components, leading to cell wall disruption and leakage of intracellular contents. Gram-positive *S. aureus* lacks an outer membrane but has a thick peptidoglycan layer rich in negatively charged teichoic acids, which promotes strong interactions with chitosan and results in effective cell wall disruption. In contrast, Gram-negative *E. coli* possesses an outer membrane that acts as a barrier, preventing chitosan from effectively disrupting the underlying cytoplasmic membrane, and under the experimental conditions used, no inhibitory effect was detected. Although chitosan with a medium molecular weight (deacetylation degree of 75%–85%) was used in this study, the observed antibacterial behavior is consistent with the literature, which shows that the antimicrobial activity of chitosan is significantly influenced by its molecular weight and degree of deacetylation. This pattern is consistent with literature reports indicating that high-molecular-weight chitosan is generally more active against Gram-positive bacteria, while activity against Gram-negative strains is limited unless low-molecular-weight chitosan is employed to penetrate the outer membrane (Goy et al., 2016; Eaton et al., 2008; Yang et al., 2018). Such a difference could be attributed to the absence of an outer membrane in *S. aureus*, which facilitates direct macromolecular damage, whereas the outer membrane of Gram-negative *E. coli* limits such destructive intracellular access. These results are consistent with previous reports and validate the necessity of AgNP incorporation for broad-spectrum antibacterial performance (Yu et al., 2025). Collectively, the data highlighted a synergistic design strategy in which AgNPs provide rapid and effective antibacterial action, AuNPs modulated host immune responses and chitosan served both as a biocompatible anchoring layer and as a secondary antimicrobial contributor against Gram-positive bacteria in acidic conditions. Importantly, the PHBV fibrous scaffold maintained structural integrity and cytocompatibility throughout functionalization, demonstrating the suitability of this biodegradable copolymer as a platform for multifunctional biomedical coatings (Guan et al., 2025; Kaya et al., 2025; You et al., 2017). The results show that the antibacterial activity of the polymer matrix with a chitosan and nanoparticle layer against *E. coli* approaches complete reduction (R ≈ 4) at concentrations of 0.1% (P/AgNP/C1) and 0.5% of chitosan (P/AgNP/C5), while at the intermediate value of 0.25% (P/AgNP/C2.5) it shows approximately half the R value. This nonlinear trend may reflect the influence of chitosan concentration on the availability and release of Ag^+^ ions from the material surface. At the lowest tested chitosan concentration (0.1%), the effect of the “bare” AgNPs predominates, with high nanoparticle availability and efficient Ag^+^ release, whereas a higher chitosan content (0.5%) can promote more intense electrostatic interactions between the positively charged amino groups and the negatively charged bacterial cell surface, leading to a synergistic antibacterial effect. At intermediate concentrations (0.25%), the polymer partially shields the NPs, reducing Ag^+^ diffusion, so the observed antibacterial effect primarily reflects the action of chitosan itself. Such nonlinear relationships between stabilizer concentration and biological effect have been repeatedly observed in polymer nanoparticle systems (Potara et al., 2011; dos Santos et al., 2021; Laanoja et al., 2025; Sherif et al., 2017). Although the biological assays were performed after the ethanol/PBS preparation procedure and therefore reflect the functional activity retained after treatment, the percentage of AgNP or AuNP loss during washing and incubation was not quantified in this study and should be addressed in future work by dedicated release/retention analyses. However, continuous immersion and repeated washing represent simplified and relatively stringent *in vitro* conditions compared with the intended topical use of the material, where the exposure environment is dynamic and depends on wound exudate, dressing replacement time, and local biological conditions.

In conclusion, the integration of anisotropic silver and gold NPs on electrospun PHBV fibers via a chitosan-mediated layer-on-layer approach yielded a multifunctional material that simultaneously addresses infection control, inflammation modulation and tissue compatibility. Such a combination of properties is rarely achieved with single-component systems and represents a significant advantage over conventional antimicrobial coatings. These findings support the potential application of the developed composites as advanced wound dressings or implantable biomaterials that can reduce infection risk while actively supporting wound healing.

## 5 Conclusion

This study successfully demonstrated the functionalization of electrospun PHBV nanofibers with silver and gold NPs via a chitosan-mediated layer-by-layer approach, achieving a well-balanced combination of antimicrobial, anti-inflammatory and biocompatible properties. The two-step synthesis of AgNPs and AuNPs produced anisotropic, colloidally stable nanostructures that were uniformly anchored onto PHBV fibers using a chitosan-based layer-on-layer approach without compromising their morphology or mechanical integrity and facilitating the stable attachment of the NPs. The antibacterial properties of the functionalized fibers were assessed against *S. aureus* and *E. coli*. Silver NPs imparted strong, broad-spectrum antibacterial activity, completely inhibiting both *S. aureus* and *E. coli*. Chitosan contributed modest antimicrobial effects against *S. aureus* and served as an efficient bioadhesive interface. The results showed that while chitosan offers modest antibacterial benefits, silver NPs are essential for a comprehensive antimicrobial response. Gold NPs enhance biological functionality by promoting anti-inflammatory responses and increasing levels of the antimicrobial peptide HBD-2. All nanocomposite fibers exhibited excellent compatibility with human keratinocytes, maintaining over 70% metabolic activity. Their biological effectiveness, strong antibacterial performance, minimal cytotoxicity and controlled immune modulation make them suitable for advanced wound dressings, antimicrobial coatings, and tissue-engineering scaffolds. Future research will aim to optimize NP loading and release kinetics, assess the long-term antibacterial effectiveness, and explore the combination of photothermal therapy and drug delivery. Overall, these findings support the development of sustainable, multifunctional biomaterials for infection control, inflammation management, and tissue repair within a single biodegradable system.

## Data Availability

The raw data supporting the conclusions of this article will be made available by the authors, without undue reservation.

## Nomenclature

PHBV: Poly(3-hydroxybutyrate-co-3-hydroxyvalerate)
**AgNO**_**3**_: Silver nitrate
AgNPs: Silver nanoparticles
AuNPs: Gold nanoparticles
**C**_**6**_ **H**_**5**_ **Na**_**3**_ **O**_**7**_ **·2H**_**2**_ **O**: Trisodium citrate dihydrate
CFU: Colony-forming units
DCM: Dichloromethane
**dH**_**2**_ **O**: deionized water
DMEM: Dulbecco’s Modified Eagle Medium
EtOH: Ethanol
HaCaT: Human epidermal keratinocyte line
**HAuCl**_**4**_ **·3H**_**2**_ **O**: Tetrachloroauric(III) acid trihydrate
HBD-2: Human β-defensin 2 peptide
IL-1α: Interleukin-1 alpha
IL-1β: Interleukin-1 beta
IL-6: Interleukin-6
IL-8: Interleukin-8
**KC**_**6**_ **H**_**7**_ **O**_**7**_ **·2H**_**2**_ **O**: Potassium dihydrogen citrate
L-L: Layer-on-layer process
MeOH: Methanol
**N**_**2**_ **H**_**4**_: Hydrazine
NaBH4: Sodium tetrahydridoborate
NaCl: Sodium chloride
**NH**_**3**_: Ammonia solution
NPs: Nanoparticles
PBS: Phosphate-buffered saline
PHAs: Polyhydroxyalkanoates
RDX: Energy-Dispersive X-ray Spectroscopy
ROS: Reactive oxygen species
SEM: Scanning electron microscopy
TEM: Transmission electron microscopy
TGF-β: Transforming Growth Factor β
TNF-α: Tumor Necrosis Factor α
UV/Vis: Ultraviolet/visible
